# Digital Behavior Change Interventions for Digital Overuse: Scoping Review of Current Approaches and Emerging AI-Enabled Systems

**DOI:** 10.2196/101150

**Published:** 2026-09-23

**Authors:** Hyeonhak Kim, Sujung Kim, Jinwoong Kim, Sangjin Park

**Affiliations:** 1 Department of Industrial Data Engineering Hanyang University Seoul Republic of Korea; 2 School of Interdisciplinary Industrial Studies Hanyang University Seoul Republic of Korea

**Keywords:** digital well-being, digital overuse, digital behavior change intervention, digital self-control tool, persuasive technology, mobile phone

## Abstract

**Background:**

Digital overuse poses a significant threat to health through multiple pathways, including the deterioration of mental health and sleep disturbance. This issue has led to the development of digital overuse-targeted digital behavior change interventions (DO-DBCIs). Although research in this field has advanced by adopting state-of-the-art technologies, substantial gaps exist in elements critical to establishing intervention validity, including target devices and activities, intervention strategies, theoretical foundations, target populations, and sustainability of effects.

**Objective:**

This scoping review aimed to systematically map DO-DBCI research across these five dimensions and to identify structural gaps and future research priorities.

**Methods:**

Following PRISMA-ScR (Preferred Reporting Items for Systematic Reviews and Meta-Analyses Extension for Scoping Reviews) guidelines, 4 databases (Web of Science, ACM Digital Library, IEEE Xplore, and PubMed) were searched on August 4, 2026, for studies published through 2025. Of 3260 records retrieved, 32 studies were included after 2-stage screening by 2 independent reviewers.

**Results:**

Pronounced structural biases were identified across all 5 dimensions. Most studies targeted smartphones exclusively (27/32, 84%) and aimed at reducing total usage time without distinguishing specific activities (26/32, 81%); no study addressed short-form video platforms. Self-monitoring was the most frequently identified strategy, followed by nudge and friction, while AI- and machine learning (ML)–based interventions remained limited. Of the 32 studies, 12 (38%) lacked an explicit theoretical framework. Target populations were heavily skewed toward young adults (17/31, 55%), with only 1 of 31 studies (3%) targeting adolescents. Most studies adopted short-term designs of 4 weeks or less, and only 38% (12/32) included any postintervention follow-up.

**Conclusions:**

This first comprehensive scoping review of DO-DBCI identifies 4 critical gaps—device and activity concentration, theory-to-design disconnect, demographic skew toward convenience samples, and insufficient longitudinal evaluation. Future research should prioritize activity-specific interventions, explicit theory-driven design, expanded population diversity, and standardized longitudinal study designs.

## Introduction

### Background

The proliferation of digital devices has expanded at an unprecedented pace over the past decade. As of 2025, approximately 5.78 billion people worldwide own a smartphone, roughly 70% of the global population, with an estimated 7.4 billion smartphones currently in use [1]. This figure represents approximately 87% of all mobile phones in circulation, with smartphones continuing to spread at an annual growth rate of approximately 3.5%. In the United States, around 91% of adults own a smartphone, and average daily online usage stands at approximately 5 hours and 16 minutes [2]. The growth of social media platforms has further accelerated this trend. The global number of social media users surged from 2.7 billion in 2017 to 5.2 billion in 2023, and average daily usage increased from 90 minutes in 2012 to 151 minutes in 2023 [3,4]. In particular, short-form video platforms, such as TikTok (ByteDance), have experienced explosive user growth from 130 million in 2018 to more than 1.7 billion in 2024, signaling a fundamental transformation in digital media consumption patterns [5].

Previous conceptual work distinguishes high levels of digital technology use from problematic use and clinically disordered use. Screen time is a quantitative indicator and does not by itself establish that use is problematic, because prolonged use may be intentional, beneficial, or contextually appropriate. Loss of control and interference with everyday functioning are important characteristics of increasingly problematic use, although these experiences may occur along a continuum rather than necessarily constituting a clinical disorder [6]. Consistent with concerns about overpathologizing everyday technology use, constructs such as problematic smartphone use (PSU) and smartphone overdependence are treated as source-specific, device-focused constructs that may overlap with, but are not synonymous with, digital overuse or addiction.

Drawing on this literature, we use digital overuse as a pragmatic, nondiagnostic umbrella term for nonclinical patterns of digital technology use that are excessive in duration, frequency, or persistence, or difficult to regulate. The term is intended as a concise and general label for what is often described in the literature as excessive screen use. It is not defined by a universal screen-time threshold or treated as a clinical diagnosis or addiction. Rather, this review focuses on everyday patterns of excessive use and difficulties in regulating digital technology use.

This rapid diffusion of digital devices has given rise to an adverse phenomenon known as “problematic use.” A meta-analysis of over 100,000 participants worldwide found that the prevalence of PSU stands at approximately 37.1%, and this figure continues to rise annually [7]. Among children and adolescents in particular, approximately 1 in 4 (23.3%) exhibit a level of PSU severe enough to impair daily functioning [8].

PSU has been associated with a range of adverse mental health outcomes. Users who experience PSU are more than 3 times more likely to exhibit depression or anxiety compared with those who do not, and the condition is closely associated with stress and diminished sleep quality [8,9]. Sleep disturbance is a multifaceted threat to user health. Smartphones not only physically displace sleep time but also disrupt circadian rhythms through the stimulating nature of content and blue light exposure [10]. PSU also has a negative effect on academic achievement, interpersonal relationships, attention, and physical health (including musculoskeletal problems and visual impairment) [11,12].

Early efforts to address the problem of problematic or excessive digital technology use relied primarily on traditional interventions delivered by health care and counseling professionals. Representative approaches included cognitive behavioral therapy (CBT), motivational interviewing, and offline “digital detox” camps. These approaches yielded effective outcomes in addressing the psychological mechanisms underlying heavy use and fostering self-insight [13,14]. However, they carry several inherent limitations. First, the absence of ecological validity means that changes achieved in controlled settings, such as clinics or camps, often fail to transfer to users’ real-world contexts [15]. Furthermore, the required involvement of professionals entails substantial time and financial constraints. In a contemporary society where digital devices have become indispensable tools for daily life and work, approaches that insist solely on absolute “disconnection” are deemed unlikely to achieve sustained long-term efficacy [6].

To overcome the limitations of traditional approaches and promote real-time behavior change within users’ daily lives, digital behavior change interventions (DBCI) have attracted considerable attention. DBCI approaches transplant intervention paradigms validated across a wide range of health behaviors, such as physical activity and smoking cessation, into digital environments [16]. More recently, active efforts have emerged to address digital overuse by viewing the interface of smartphones or PCs as the medium of intervention, targeting the very microcontext in which problematic behavior occurs. The domain of DBCIs specifically designed to address excessive use is known as digital overuse-targeted DBCI (DO-DBCI).

Research in the DO-DBCI field has developed primarily around tools that help users self-regulate their device use, commonly referred to as digital self-control tools (DSCTs). Initial approaches were dominated by restrictive strategies, such as usage time limits and app-launch blocking. GoalKeeper (developed by Kim et al [17]) provided a mechanism that progressively extended lockout durations or imposed all-day blocking once a daily usage threshold was exceeded. Alternatively, PomodoLock (developed by Kim et al [18]) applied the Pomodoro technique to simultaneously block disruptive elements on both PCs and smartphones during focus periods. Although these tools pursued immediate suppression effects, they were criticized for violating user autonomy through coercive methods and for failing to maintain behavioral change after the intervention ended.

In response to these limitations, a diverse range of noncoercive intervention strategies grounded in behavioral science theory emerged. Nudge-based interventions guide users toward desirable behaviors without compelling specific choices. Chai Wallpaper (developed by Nwagu and Orji [19]) applied ambient feedback by animating falling leaves on the wallpaper proportional to usage time, while Okeke et al [20] delivered subtle vibrations during overuse to allow users to naturally self-monitor. Brockmeier et al [21] adopted a self-nudging approach that displayed full-screen prompts during Instagram (Meta) use, allowing users to decide for themselves whether to stop.

Friction-based interventions insert intentional supplementary tasks before app access, using the perspective of dual process theory [22] to shift automatic usage (system 1) toward deliberate judgment (system 2). LocknType (developed by Kim et al [23]) raised the interaction cost of use by requiring the entry of random numbers at app launch, while TypeOut (developed by Xu et al [24]) advanced this approach by having users type self-affirmation phrases, combining friction with cognitive intervention. This friction-based approach extended beyond software interfaces into physical forms.

The scope of interventions also expanded into the domains of self-awareness and self-monitoring. TimeAware (developed by Kim et al [25]) classified PC usage time as productive or unproductive and visualized it with positive or negative framing, thereby altering how office workers perceived productivity. MeTime (developed by Whittaker et al [26]) promoted continuous awareness through an always-on display widget that showed real-time usage durations in the corner of the PC screen. MindsCare (developed by Lee et al [27]) took a more comprehensive approach, implementing a health care platform that collected and analyzed smartphone usage data to assess overdependence risk and link this to clinical feedback, demonstrating the potential to connect self-monitoring with expert mediation. Meanwhile, Aiki (developed by Inie and Lungu [28]) attempted a distinctive redirection strategy; when users attempted to visit time-wasting sites, the system intercepted and presented brief language-learning quizzes, converting unproductive time into learning opportunities.

Interventions using social context formed another meaningful strand of research. NUGU (developed by Ko et al [29]) proposed a social comparison mechanism in which group members shared usage-restriction goals and were ranked against one another. Lock n’ LoL (developed by Ko et al [30]) introduced a colocation-based collective restriction mechanism whereby individuals present in the same physical space jointly locked their smartphones, suggesting that social bonds can reinforce self-regulation. FamiLync (developed by Ko et al [31]) extended this social approach to the family unit, exploring the possibility of family-level mediation through a dashboard that enabled parents and children to share each other’s app usage data.

Incentive-based interventions use monetary or nonmonetary rewards and penalties to reinforce reduced usage. GoldenTime (developed by Park et al [32]) introduced a loss framing strategy by assigning timeboxing goals and deducting accumulated credits upon failure, thereby inducing behavior change.

The intervention strategies used in these studies varied considerably. Keller et al [33] proposed a mobile app grounded in the health action process approach to train self-efficacy and planning competencies. Kent et al [34] evaluated a smartphone-delivered intervention combining goal setting, personalized feedback, mindfulness, and behavioral suggestions among 10 students. Additionally, Hamamura et al [35] reported that nudge interventions combining focus features and social comparison could reduce PSU among adolescents.

The most recent technological advances involve the emergence of personalized and adaptive interventions incorporating AI and machine learning (ML). Time2Stop (developed by Orzikulova et al [36]) implemented a just-in-time adaptive intervention (JITAI) that predicts the optimal timing of interventions using ML, while integrating explainable AI (XAI) to enhance system transparency and user acceptance. Jang et al [37] demonstrated further technological progress by using a reinforcement learning (RL) algorithm (Thompson sampling) to predict individual response probabilities and dynamically compute the magnitude of the optimal monetary reward. This suggests that the DO-DBCI field is evolving from static, one-size-fits-all interventions toward intelligent systems that respond in real time to users’ contexts and behavioral data.

### Previous Reviews and Research Gaps

The rapid growth of DO-DBCI articles has been accompanied by an accumulation of review studies aimed at providing comprehensive perspectives on this literature. In the early phase, Lyngs et al [38] classified and evaluated 367 commercial digital self-control tools available in major app stores using a framework grounded in the dual process theory of cognitive neuroscience, providing an overview of the tools available in the market. Building on this market-centered analysis, Biedermann et al [39] systematically examined the effectiveness of digital self-control interventions aimed at reducing digital distractions. Subsequently, Monge Roffarello and De Russis [40] synthesized over a decade of academic discourse through a systematic review and meta-analysis, comprehensively analyzing the motivations, design strategies, and evaluation methods of DSCTs, thereby providing the most extensive overview of the field to date.

Previous reviews have substantially advanced research on digital self-control by examining commercial tools, intervention effectiveness, and the broader DSCT literature. As summarized in Table 1, this review extends this body of work through a multidimensional scoping synthesis of functioning digital interventions specifically targeting digital overuse. It jointly examines target devices and activities, intervention strategies and technical modalities, theoretical bases and frameworks, target populations, and intervention duration and sustainability, with particular attention to how theoretical foundations are translated into intervention design. The search coverage through 2025 additionally captures recent developments in AI-, ML-, JITAI-, XAI-, and reinforcement learning–based adaptive interventions.

**Table 1:** Comparison of previous reviews and this scoping review.

Aspect	Lyngs et al [38]	Biedermann et al [39]	Monge Roffarello and De Russis [40]	This review
Scope	Commercial digital self-control tools	Digital self-control interventions	Digital self-control tools	Digital overuse-targeted DBCIs^a^
Corpus	367 commercial tools	16 publications	62 publications	32 publications
Analytical focus	Design features, intervention strategies, and underlying self-regulation mechanisms	Intervention features, outcomes, effectiveness, and study quality	Motivations, strategies, theories, challenges, ethics, evaluation, and effectiveness	Devices and activities, strategies and technologies, theories, populations, and sustainability
Unique contribution	Theory-based functionality taxonomy of commercial DSCTs^b^	Effectiveness synthesis of evaluated DSCT interventions	Comprehensive DSCT synthesis and meta-analysis of technology-use reduction	Multidimensional mapping of DO-DBCI^c^ research and theory-to-design translation

^a^DBCI: digital behavior change intervention.

^b^DSCT: digital self-control tool.

^c^DO-DBCI: digital overuse-targeted digital behavior change intervention.

Despite the expansion of research and technological advances in this field, the current DO-DBCI evidence base continues to exhibit 4 major limitations. These limitations can be summarized under the following four key headings: (1) concentration on a narrow range of target devices and behaviors, (2) insufficient translation of theoretical constructs into intervention design, (3) demographic bias in target populations, and (4) limited evidence for long-term efficacy and sustainability of behavior change.

First, the approach to target devices and behaviors is heavily biased toward a single framing, that is, reducing the “total screen time” of smartphones. Digital overuse should be understood not merely as a device-level problem but as a phenomenon that occurs at the level of specific online activities. For instance, just as the *ICD-11* (*International Classification of Diseases 11th Revision*) categorized gaming disorder as an independent diagnosis (World Health Organization, 2019), gaming, social media, and short-form video each possess distinct usage motivations and psychological mechanisms [41,42]. In particular, problematic use of short-form platforms, such as TikTok, is driven by platform-specific design affordances, such as infinite scroll and immediate reinforcement, emphasizing the need for activity-specific interventions distinct from those used for traditional media [43]. Furthermore, real-world overdependence is not confined to a single device; it persists through cross-device switching among smartphones, PCs, and tablets. Nevertheless, most existing research focuses exclusively on smartphones, and integrated intervention strategies encompassing multidevice environments remain limited [38,44].

Second, while most studies cite various behavioral science theories, such as dual process theory and nudge theory, very few (eg, studies by Xu et al [24] and Park et al [32]) provide empirical evidence of how the core constructs of these theories have been translated into the specific functions of the intervention system. For example, studies that cite self-determination theory as a rationale frequently fail to explain how the core needs of autonomy, competence, and relatedness were operationalized in the design, making it difficult to verify the mechanisms of intervention theoretically.

Third, there is a demographic bias in target populations (related to research question [RQ] 4). Existing studies continue to rely heavily on convenience sampling of university students who are readily accessible. Despite adolescents being the most vulnerable population to indiscriminate digital device use due to their developing cognitive self-regulation capacities, studies that directly target this group and validate the efficacy of personalized interventions are extremely rare [35,45], revealing a serious research gap.

Fourth, evidence for long-term efficacy and the sustainability of behavior change remains limited. Because patterns of digital overuse may persist or recur after a short-term intervention, sustained behavior change requires longer-term evaluation. However, the majority of existing studies are limited to short-term experiments lasting only a few weeks, and studies that systematically address the long-term efficacy and sustainability of behavior change are absent.

### This Study and RQs

To address the 4 gaps outlined above comprehensively, an approach is needed that goes beyond meta-analyses that aggregate effect sizes from individual studies or systematic reviews limited to specific contexts. This study aims to restructure fragmented data within an integrative analytical framework, thereby supplementing the limitations of existing research and systematically identifying macro-level gaps across the field. Accordingly, this review maps the characteristics of the existing DO-DBCI literature across the following 5 key dimensions: target (device and behavior), strategy, theory, population, and sustainability of effects. This multidimensional analysis provides macro-level insights that are not confined to the figures of individual studies, serving as a decisive foundation for presenting a systematic roadmap for future research. To this end, this scoping review addressed the following 5 specific RQs.

- RQ1: What devices and target activities have DO-DBCI studies addressed?
- RQ2: How have DO-DBCIs been implemented in terms of intervention strategies and technical modalities?
- RQ3: What theoretical foundations have DO-DBCI interventions been designed upon?
- RQ4: In what target populations have DO-DBCI studies been conducted?
- RQ5: What are the durations of DO-DBCI interventions, and how has the sustainability of intervention effects been evaluated?

The Methods section describes the literature search and selection methodology of this scoping review, while the Results section presents the analytical results for the 5 RQs based on the collected literature. Discussion section synthesizes the findings of this review to discuss future research directions for overcoming the limitations of the DO-DBCI field, and the Conclusion section presents the conclusions of this study.

## Methods

### Overview

This study is a scoping review conducted in accordance with the PRISMA-ScR (Preferred Reporting Items for Systematic Reviews and Meta-Analyses Extension for Scoping Reviews) [46] guidelines. The completed PRISMA-ScR checklist is provided in Multimedia Appendix 1. No protocol for this scoping review was registered a priori. The purpose of this review is to systematically explore existing research on DO-DBCI and to structurally map the research landscape spanning theoretical foundations, intervention strategies and technical design, target populations and research contexts, measurement indicators and analytical methods, research design and intervention duration, and existing research gaps.

Following the PRISMA-ScR guidelines, this study systematically searched and screened relevant literature, extracted key information, and synthesized the results to identify research trends and future research opportunities.

The DO-DBCI field spans multiple disciplinary backgrounds, including psychology, human-computer interaction (HCI), public health, and computer science, and exhibits high heterogeneity in the theories used, intervention strategies, technical modalities, and target populations. Scoping reviews are a useful approach in heterogeneous and fragmented research fields such as this. Rather than drawing a single conclusion to a specific RQ, they systematically map the scope, methodological diversity, and areas requiring further research.

### Search Strategy

The following four academic databases were used as information sources: (1) Web of Science, (2) ACM Digital Library, (3) IEEE Xplore, and (4) PubMed. ACM Digital Library and IEEE Xplore were selected as core resources in the HCI and computing fields, covering system design and user evaluation; PubMed was included for health behavior and clinical intervention research; and Web of Science was chosen to cover multidisciplinary literature spanning psychology and behavioral science. This combination reflects the multidisciplinary character of DO-DBCI, which spans HCI, psychology, public health, and behavioral science.

Search terms were constructed by consulting search strategies from available review studies [38-40] and composing Boolean combinations of 2 core concepts—digital overuse and behavior change intervention. Terms related to digital overuse included “computer usage,” “digital wellbeing,” “digital overuse,” “digital distraction,” “digital dependence,” “gaming disorder,” “internet overuse,” “mobile phone overuse,” “mobile device overuse,” “mobile distraction,” “off-task usage,” “smartphone overuse,” “smartphone usage,” “smartphone use,” “smartphone non-use,” “smartphone app use,” “smartphone addiction,” “smartphone distraction,” “social media usage,” “time-wasting websites,” and “time online,” combined with the OR operator. Terms related to behavior change interventions included “behavior change,” “behavior restriction,” “intervention,” “limiting behavior,” “persuasive technology,” “self-control,” “self-discipline,” “self-monitoring,” “self-regulation,” and “time application,” combined with the OR operator. These 2 sets were connected with AND. Search fields (title, abstract, and keywords) and query formats were adjusted to match the interface of each database, and publication year was set from the earliest searchable date to 2025. The final search was conducted on August 4, 2026. The complete database-specific search strategies and the number of records retrieved from each database are provided in Multimedia Appendix 2.

### Screening

Literature screening was conducted in the following 3 stages: identification, screening, and inclusion. During the screening stage, inclusion and exclusion criteria were applied sequentially across 2 steps, that is, title and abstract screening, followed by full-text review. Screening was performed independently by 2 researchers (HK and SK), with disagreements resolved through consensus. The number of studies at each stage of the screening process is summarized in the PRISMA flowchart. The eligibility criteria applied during screening are presented in Textbox 1.

**Inclusion and exclusion criteria.**

**Inclusion criteria:**

- Studies that designed, developed, or evaluated a functioning system aimed at reducing problematic or excessive use of digital devices (smartphones, PCs, tablets, etc)
- Written in English
- Published in a peer-reviewed outlet
- Investigated a nonclinically recruited population, meaning that participants were neither recruited as patients through healthcare or counseling settings nor required to have a clinician-confirmed diagnosis.

**Exclusion criteria:**

- Studies using only face-to-face counseling, pharmacotherapy, or traditional psychotherapy without a digital intervention component
- Studies in which reducing problematic or excessive digital-device use was only a secondary or incidental function rather than the primary intervention aim.
- Studies conducting only surveys or observational assessments without system design or development
- Study protocols that did not report empirical results from a completed study
- Theses, conference abstracts, editorials, book reviews, and gray literature
- Studies for which full-text access was unavailable

### Data Charting

Key information was extracted and analyzed from the included studies using a structured data extraction form. Extraction items were designed to correspond to RQ1-RQ5. In addition to basic information (authors, publication year, country, and tool name), the following analyses were conducted. For RQ1, target behaviors and devices of each intervention were extracted, and frequency distributions were analyzed. For RQ2, intervention strategy types and technical modalities, as well as the use of AI, ML, and XAI, were extracted and analyzed for frequency distribution and usage status. For RQ3, explicitly cited theories and the method by which these theories were translated into design features were extracted, and frequency analysis by theory and cross-analysis between theory and strategy were conducted. For RQ4, participant age groups, gender, sample size, and country of study were extracted, and their distributions were analyzed. For RQ5, intervention duration and presence of post-intervention follow-up measurement were extracted and summarized. In addition, author-reported limitations and recommendations for future research were recorded. Extraction results are summarized in Multimedia Appendix 3 [17-21,23-37,47-58], and analytical results are presented visually through charts and tables alongside a narrative description.

## Results

### Study Selection

This section presents the results of the identification, screening, and inclusion of studies within the scope of the research objectives. During the identification stage, the search strategy described in the Methods was applied to retrieve a total of 3260 records from 4 academic databases (Web of Science: n=2029, ACM Digital Library: n=125, IEEE Xplore: n=166, and PubMed: n=940). After removing 930 duplicate records, 2330 studies entered the screening stage.

During the screening stage, the screening of titles and abstracts was first conducted on 2330 records. In accordance with the established inclusion and exclusion criteria, 2217 records were excluded (ie, records not addressing technology-based interventions aimed at reducing digital overuse; secondary literature, such as reviews, meta-analyses, and commentaries; and records outside the scope of this review). The remaining 113 records underwent full-text screening. Upon reviewing the full texts of these 113 records, an additional 81 were excluded for other reasons, including not incorporating the design or evaluation of an intervention system, targeting health behaviors other than digital overuse, and requiring a special hardware device for the intervention. As a result, 32 studies [17-21,23-37,47-58] were confirmed as the analytical corpus for this scoping review. The paper selection process is summarized in Figure 1.

**Figure 1:**
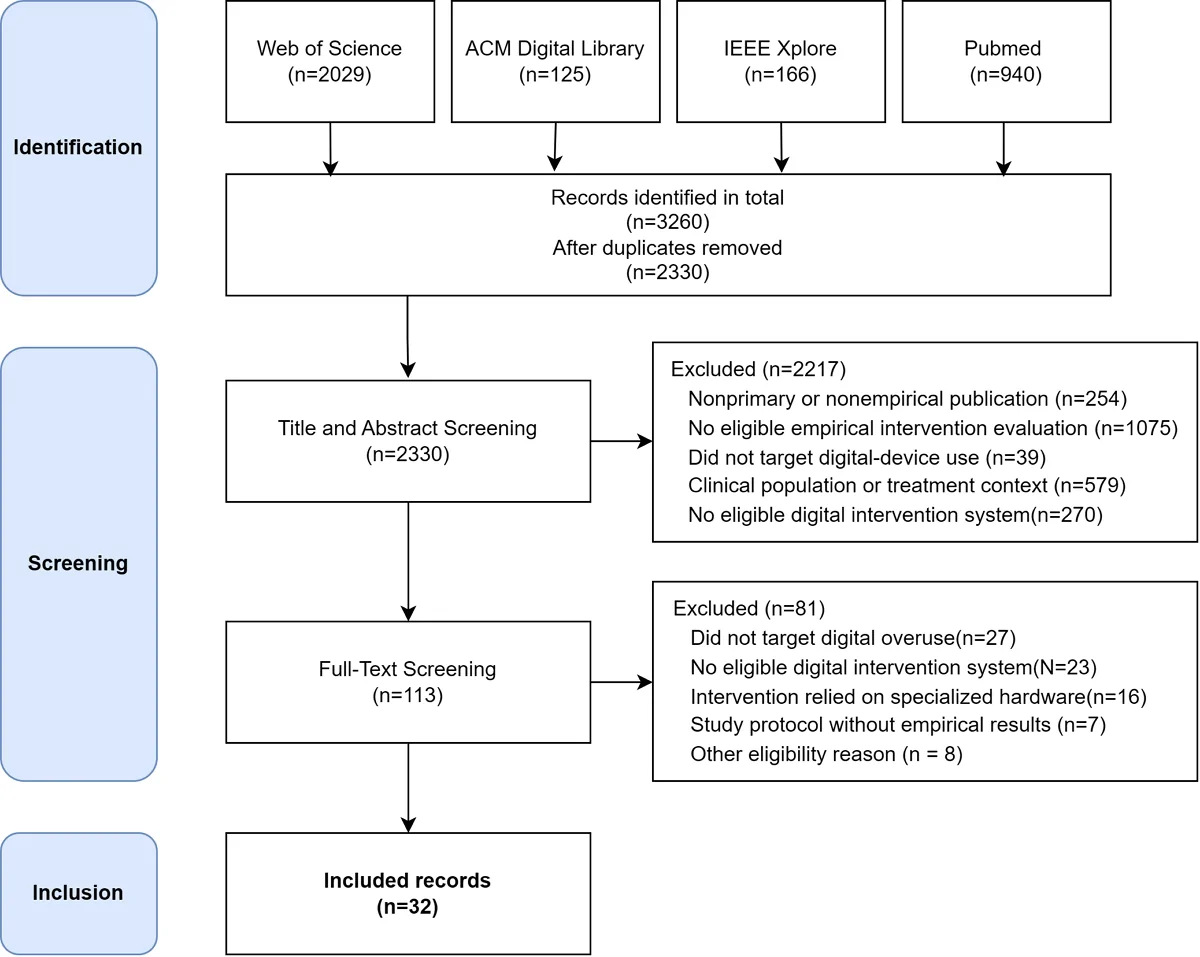
PRISMA (Preferred Reporting Items for Systematic Reviews and Meta-Analyses) flowchart of the corpus selection process.

### RQ1: Target Devices and Activities

Analysis of the devices and activities targeted by the interventions in the included studies revealed a pronounced concentration across both device and activity dimensions.

When the included studies were classified by target device, single-device–centered interventions were found to be dominant. Specifically, studies targeting smartphones as a sole platform accounted for the overwhelming majority at 84% (27/32), followed by studies focusing on the PC environment at 13% (4/32). By contrast, studies that comprehensively addressed multidevice user environments, simultaneously controlling both smartphones and PCs, accounted for only 3% (1/32), clearly demonstrating the skewed predominance of single-device interventions.

A similar concentration was observed for target behavior (activity). Among all studies, 81% (26/32) targeted “general (non–activity-specific) smartphone or PC use” without distinguishing specific apps or activities, aiming to reduce total usage time or the number of unlocks. In other words, most analyzed studies adopted an inclusive approach that does not differentiate between goal-directed and habitual use. The remaining 19% (6/32) targeted specific activities; 5 studies focused on social media [20,21,47-49], while 1 targeted video streaming [50]. That social media constituted the majority of activity-specific interventions and serves as the most prominent target is attributable to the unique addictive design features of these platforms, such as infinite scroll, unpredictable rewards, and social validation, which most powerfully trigger mindless, habitual engagement.

### RQ2: Intervention Strategies and Technical Modalities

#### Overview

This section classifies the intervention strategies used for DO-DBCI by type. It then surveys the distribution and key characteristics of each strategy. Finally, it analyzes the overall evolutionary trajectory of technical modalities used to deliver interventions, ranging from simple software control to sensor-based context awareness, physical and ambient interfaces, and ultimately, intelligent adaptive systems.

Concepts such as “nudge” and “CBT” among the intervention elements analyzed in this review occupy a dual status, both as technical methods (intervention strategies) that drive behavior change and as academic foundations (theoretical foundations) that explain those methods. Accordingly, in this report, these concepts are discussed as “strategies” when focusing on technical implementation, and as “theories” when addressing the logical rationale underlying design.

#### Intervention Strategies

When the intervention components used in the 32 included studies were classified at the level of individual behavior-change techniques, 11 strategy types were identified. Because a single study could use multiple strategies, the categories were not mutually exclusive. Self-monitoring was the most frequently adopted strategy (n=10), followed by nudge (n=6) and friction (n=5). Block or removal, social support, and goal setting or advancement were each identified in 4 studies. Less frequently adopted strategies included incentive (n=2), psychological skills training (n=2), punishment (n=1), mindfulness (n=1), and persuasion (n=1).

Self-monitoring strategies promote self-awareness and self-regulation by objectively showing users their usage patterns. This category includes productivity-framing visualization [25], big-data–based diagnostic reports [27], always-on display widgets [26], ambient feedback [19], and gamification-based self-tracking [51]. Particularly noteworthy among these studies was TimeAware [25], which went beyond simply displaying usage time by framing and visualizing device use as “productive time” and “interrupted time,” thereby stimulating users’ motivation for behavior change.

Nudge interventions are soft intervention methods that help users voluntarily change their behavior by naturally drawing their attention without forcibly blocking device use. In digital environments, they take the form of covertly transforming the digital interface environment through vibration, visual changes, and similar stimuli. The nudge strategies identified in this review encompass diverse subtypes, including pop-up notifications [21,49], haptic feedback [20], periodic visual feedback [48], and multicomponent changes to smartphone settings and the surrounding environment [52]. For instance, Okeke et al [20] induced awareness without interrupting the user’s workflow by delivering subtle haptic feedback when usage exceeded a threshold. Nwagu and Orji [19] applied an ambient nudge in which leaves on the wallpaper fell in proportion to smartphone usage time, providing ambient visual feedback on accumulated use.

Friction-based interventions (n=5), meanwhile, impose intentional cognitive or physical costs on users while allowing access to remain possible. Representative examples of cognitive friction include LocknType [23], which requires entry of random numbers before app launch, and TypeOut [24], which has users type self-affirmation phrases aligned with their values. Other approaches introduced friction by redirecting users from time-wasting websites to microlearning activities [28], requiring a typing task at adaptively selected intervention moments [36], or modifying touchscreen gestures after a usage threshold was exceeded [53].

Block or removal strategies (n=4) directly restricted access to distracting apps or removed potentially distracting interface elements. GoalKeeper [17] locked users into self-defined daily usage limits, while PomodoLock [18] temporarily blocked selected apps and websites across PCs and smartphones during synchronized focus sessions. At the feature level, the intervention developed by Lyngs et al [47] removed the Facebook (Meta) newsfeed, whereas SwitchTube (developed by Lukoff et al) [50] provided a focus mode in which recommendation-based features were removed from the video-streaming interface.

Social support strategies promote individual behavior change within relational contexts with others and were implemented through peer usage comparison [29,35], mutual family mediation [31], and collaborative device lockout [30]. Among these diverse approaches, NUGU [29] induced social comparison through usage-restriction rankings among group members, while Lock n’ LoL [30] facilitated distraction-free interaction by having members simultaneously lock their smartphones during in-person gatherings.

The remaining strategies appeared less frequently. Incentive-based interventions (n=2) provided financial rewards contingent on reduced smartphone use [32,37], whereas punishment (n=1) involved deducting accumulated credits when hourly usage goals were not met in GoldenTime [32]. Psychological skills training (n=2) used structured exercises addressing goal setting, mindfulness, planning, and behavioral skills [33,34]. Mindfulness (n=1) was operationalized in MindPhone (developed by Terzimehić et al [54]) through reflective prompts presented when users unlocked their smartphones, while persuasion (n=1) was implemented in MindShift (developed by Wu et al [55]) through personalized messages generated from users’ usage context, goals, habits, and self-reported mental states.

#### Technical Modalities

This section analyzes the technologies through which the intervention strategies described above are implemented, according to the “evolution of technical complexity.” In terms of technical modality, a clear trajectory of technological evolution was evident, from simple software control to AI-powered adaptive systems.

The most basic technical approach is “static control and friction.” This involves using the basic control permissions of the operating system (OS) or browser to physically block or delay user access. In PC environments, representative examples include Hack Myself [47], which directly manipulates document object model elements to permanently remove newsfeeds, and PomodoLock [18], which scans background processes and forcibly terminates distracting apps (process killing). On mobiles, technical approaches include cognitive friction techniques [23,24], which intercept app-launch events (input interception) and require the entry of random numbers or self-affirmation phrases, and Aiki [28], which forcibly redirects (URL interception and redirection) users attempting to access specific URLs to quiz pages. Unlike interventions triggered when users launch an app or access a website, InteractOut (developed by Lu et al [53]) introduces interaction friction during app use by remapping common touchscreen gestures after a predefined usage threshold is exceeded.

To overcome the limitations of simple blocking, this evolved into “context-awareness and automation.” Rather than unconditionally blocking user access, this approach intelligently identifies “situations requiring intervention” by using sensors built into the device or system functions. Lock n’ LoL (developed by Ko et al [30]) uses Wi-Fi scanning and colocation technology to detect when group members are physically gathered together and initiates synchronized group locking. Progress was also made by leveraging automation tools natively provided by the smartphone OS without developing separate background apps. MISFEED (developed by Purohit and Holzer [48]) implements nudge automation by leveraging the iOS “Shortcuts” (Apple Inc) feature to deliver automated visual banners that provide real-time feedback on usage time when a social media app is launched.

Further physical expansion into “ambient and haptic interfaces” occurred to reduce interaction fatigue within the smartphone screen. Chai Wallpaper [19] showcased ambient data visualization technology that combined screen-on time with physical activity sensor data to cause a wallpaper tree to change in real time. The intervention developed by Okeke et al [20] used a haptic feedback interface that continuously generates only subtle vibrations when a limit is exceeded, without visual intervention.

The most advanced technological evolution currently occurring in the DO-DBCI field is the introduction of “AI-driven adaptive systems.” Going beyond passively responding to fixed rules or sensor values, these systems use ML algorithms to learn from individual user data and evolve autonomously. Time2Stop [36] implements a JITAI system that predicts the “optimal timing” of interventions through ML-based adaptive user modeling, while introducing XAI to explain the AI decision-making process and enhance user trust in the system. Additionally, Jang et al [37] demonstrated technological advancement by adopting a Thompson sampling–based RL algorithm to predict individual response probabilities and dynamically compute the optimal monetary reward magnitude. In addition, MindShift [55] used an LLM to generate personalized intervention messages based on users’ usage contexts, goals, habits, and self-reported mental states.

Taken together, the analytical results show that DO-DBCI research has evolved into a diverse array of technical forms encompassing AI and various physical interfaces, beyond simple software control. However, for these technological advances and diverse intervention strategies to lead to genuine long-term behavior change in actual users, a robust theoretical foundation grounded in behavioral science (theory-driven design) should be provided, going beyond mere functional implementation. No matter how sophisticated the system is, indiscriminately combining individual features without a clear understanding of users’ cognitive and behavioral change mechanisms can provoke psychological resistance or result in effects that remain only temporary. The RQ3: Theoretical Foundations section examines the specific theoretical backgrounds in psychological and behavioral science, upon which the interventions described above were designed.

### RQ3: Theoretical Foundations

Of the 32 included studies, 20 (63%) explicitly reported at least 1 theory, model, or framework as a basis for the intervention, whereas 12 (38%) did not report an explicit theoretical foundation. Only theories, models, or frameworks explicitly identified by the study authors as informing the intervention were included in this classification.

The reported foundations were highly heterogeneous, encompassing more than 20 distinct theories, models, and frameworks from psychology, behavioral science, human-computer interaction, and adaptive intervention research. Moreover, 4 recurring foundations or theoretical families and their applications are discussed further in this study in greater detail.

First, dual process theory and dual systems theory were grouped into a single theoretical family because both distinguish between automatic or impulsive processes and reflective or deliberative processes, despite differences in terminology and specific theoretical formulations. In Kahneman’s formulation, dual process theory [22] explains human cognition in terms of “system 1,” which operates automatically and impulsively, and “system 2,” which engages in deliberate and rational control. Studies drawing on these perspectives characterized habitual smartphone checking and aimless scrolling as behaviors driven primarily by automatic processes and sought to engage reflective control. Some studies operationalized this approach by introducing friction at app launch or during continued use [23,24,36,53], whereas others modified or restricted the digital environment or provided context-sensitive persuasive support [17,47,50,55].

Second, self-regulation theory [59] explains that humans control their behavior through a continuous feedback loop of “goal setting→self-monitoring→self-evaluation.” Interventions based on this theory provide intuitive visualizations of usage data so that users can objectively confront their habitual smartphone usage [19]. In a related approach, by visually showing users the gap between their self-set goals and actual usage, this theory stimulates intrinsic motivation for behavioral self-correction [29,32].

Third, social cognitive theory [60] and social comparison theory [61] hold that the process of observing the behavior of others and comparing it to one’s own serves as a powerful catalyst for behavior change. Studies adopting this perspective transform digital overuse from a matter of individual willpower to be overcome alone into a challenge that can be addressed within social relationships. These theories play a central role in generating constructive forms of peer pressure and social support by having users share usage-restriction goals with family or peers and compare each other’s achievement levels [29,31].

Fourth, nudge theory [62] constitutes the behavioral economics foundation for the nudge interventions discussed in the RQ2: Intervention Strategies and Technical Modalities section. This theory explains how human decision-making changes in predictable directions according to the design of the surrounding environment, or “choice architecture.” As explained in the preceding section on nudge as an intervention strategy, and in line with the basic premises of nudge theory, interventions using nudge theory modify the user’s environment (here, ambient functions of digital devices) to change behavior (through noncoercive means) [20,35,56]. This approach focuses on gently guiding users to stop usage of their own volition, without resistance.

Other reported foundations included CBT, the transtheoretical model (TTM), acceptance and commitment therapy, the health action process approach, existence, relatedness, and growth (ERG) theory, choice architecture, and persuasive technology design principles [33,55-57]. However, the degree to which core constructs were explicitly and systematically translated into actual design features (translation) varied considerably across studies, even among those using the same theory. An in-depth analysis of the connectivity between theory and technical implementation, and its limitations, is discussed in the Discussion section.

### RQ4: Populations

When the target populations were classified by age, young adults accounted for the largest proportion at 55% (17/31), followed by adults at 26% (8/31), mixed populations at 16% (5/31), and adolescents at 3% (1/31). In this review, each group was operationally defined as follows: adolescents as the underage group in the 12-17 age range, young adults (including university students) as the early adulthood group primarily in the 18-30 age range, adults as the broader adult population aged 18 and older, and mixed populations as samples spanning multiple age or social groups or not assignable to a single age group. This distribution indicates that DO-DBCI research is generally concentrated on early adulthood and adult populations, while research targeting adolescents and general users was relatively limited.

Analysis of gender distribution revealed that only 69% (22/32) of the included studies clearly reported gender information, indicating incomplete reporting of basic demographic characteristics. Aggregating participants across these 22 studies, the overall gender distribution was approximately balanced, with 49% (1291/2612) male and 51% (1321/2612) female participants. However, this aggregate balance masked substantial variation across individual studies. To characterize this study-level variation, we calculated, for each study, the percentage of participants belonging to the more numerous gender. This percentage ranged from 52% to 100%, with a median of 64%. Moreover, in 45% (10/22) of the studies, one gender was represented at least twice as often as the other, including 2 studies that included participants of only one gender. Thus, the approximately balanced pooled distribution should not be interpreted as indicating consistently balanced gender representation across individual studies. The incomplete reporting of gender further limits systematic assessment of demographic representativeness across the DO-DBCI evidence base. Given the generally small sample sizes of the included studies, these gender-distribution findings should be interpreted descriptively and not as evidence of gender-related differences in intervention effects.

Sample sizes ranged from 10 to 976, with a mean of 95 (SD 175) and a median of 46 (IQR 27-71). When sample size was categorized as small (n≤50), medium (50<n≤200), or large (n>200), small-scale studies were most common at 58% (18/31), followed by medium-scale at 29% (9/31) and large-scale at 13% (4/31). Furthermore, 1 study did not report the sample size and was therefore excluded from the sample-size analysis. In total, 87% (27/31) of the studies were conducted with samples of fewer than 200 participants, indicating that DO-DBCI research is generally based on small- to medium-scale studies.

In summary, DO-DBCI research is characterized by the following 3 structural features: age concentration centered on university students and young adults, a prevalence of small- to medium-scale samples, and limited reporting of gender information.

### RQ5: Intervention Duration and Sustainability of Effects

The experimental designs of DO-DBCI studies generally follow a 3-stage structure: baseline–intervention–follow-up. This study analyzed the distribution of durations at each stage by distinguishing between AI- and ML-based and Heuristic-based interventions. Among the 32 included studies, 4 were classified as AI- or ML-based interventions and 28 as Heuristic-based interventions. Accordingly, the subgroup comparison included all 32 studies. AI- and ML‑based approaches learn intervention mechanisms through algorithms such as contextual ML and RL to determine optimal intervention timing and intensity, whereas Heuristic-based interventions apply fixed or predefined intervention logic without data-driven adaptation. Overall, 56% (18/32) of the studies had total study durations of 4 weeks or less. This short-duration pattern was more pronounced among Heuristic-based studies, with 61% (17/28) having total study durations of 4 weeks or less, compared with 25% (1/4) of AI/ML-based studies. The distributions of baseline, intervention, and follow-up durations across all included studies are summarized in Table 2.

**Table 2:** Distribution of baseline, intervention, and follow-up durations across included studies.

Study stage and duration category		Studies, n (%)
Baseline		
	Not conducted or not reported	8 (25)
	1 week	19 (59)
	2 weeks	5 (16)
Intervention		
	1 week	5 (16)
	2-3 weeks	19 (59)
	4-5 weeks	5 (16)
	≥6 weeks	3 (9)
Follow-up		
	Not conducted	20 (63)
	1 week	3 (9)
	2-3 weeks	7 (22)
	≥4 weeks	2 (6)

The baseline stage was used to collect data on users’ daily behavioral patterns before intervention, establishing a reference point for comparison with postintervention changes. In AI- and ML-based studies, it also served as an initial data collection period for building personalized predictive models. Studies that explicitly reported the baseline accounted for 75% (24/32) of the total, while the remaining 25% (8/32) either did not include this stage or did not report relevant information. In both types, baseline duration tended to be short, averaging approximately 1 week.

Intervention duration, the stage during which technical and psychological interventions were applied to participants to induce behavior change, ranged from 0.5 weeks to a maximum of 8 weeks. Analysis by intervention type revealed that Heuristic-based studies most frequently adopted short durations centered on 1-2 weeks (16/28, 57%), whereas AI- and ML-based studies showed a relatively higher tendency to apply longer intervention durations of 3 weeks or more (3/4, 75%).

The follow-up stage involves observing whether the intervention effects persist after the intervention has fully concluded. Only 38% (12/32) of the studies conducted a follow-up, whereas the remaining 63% (20/32) concluded at the end of the intervention stage without any follow-up assessment. Among the 12 studies that conducted a follow-up, 75% (9/12) adopted a short-term follow-up period of 1-2 weeks. By intervention type, Heuristic-based studies accounted for the majority of studies conducting a follow-up, representing 83% (10/12). Among these, 80% (8/10) adopted a short-term follow-up period of 1-2 weeks.

Overall, it was confirmed that DO-DBCI research is skewed toward “short-term observation-centered experimental designs.” This design pattern differs from other domains of behavior change intervention research. For example, in smoking cessation behavior intervention research, follow-up observations lasting several months or more are standard for evaluating the maintenance of intervention effects and risk of relapse. Some smoking cessation studies apply long-term tracking designs that assess the sustainability of behavioral change up to 52 weeks following interventions lasting 24 weeks or more [63,64]. Furthermore, in personalized digital interventions, the need to accumulate and analyze user data over extended periods has been emphasized, as user behavior patterns and engagement trajectories change over time [65,66]. From this perspective, personalized interventions require not only a follow-up to evaluate postintervention effectiveness but also a sufficiently extended baseline observation period and intervention period within which individualized strategies are applied. However, the DO-DBCI studies identified in this review tended to adopt comparatively short experimental designs across all of these stages. Studies that systematically evaluated the maintenance of behavior change and the effects of personalized intervention over extended periods remain limited.

The key research gaps and limitations derived from the analytical results for RQ1-RQ5 above are interpreted in depth in Discussion, which connects them to specific recommendations for future research directions.

## Discussion

This section provides an in-depth interpretation of the results for each RQ, together with integrated implications, limitations, and directions for future research in the DO-DBCI field.

### RQ1: Bias in Target Behavior and Device and the Need for Expansion

The results of this review demonstrate that the devices and behaviors targeted by DO-DBCI research are heavily concentrated in specific areas. From the perspective of the target device, most studies (31/32, 97%) remain confined to controlling only a single device, either a smartphone or a PC, and only 1 study (3%) considered multidevice environments. This single-device–centered approach has clear structural limitations in view of contemporary digital lifestyles. It cannot effectively counter the so-called “balloon effect,” whereby a user whose access to a specific app is blocked on a smartphone continues to access it via PC or tablet, nor can it prevent usage displacement, in which restricting use on one device triggers a transfer to another. In this context, PomodoLock [18], which integrated real-time cross-device synchronization between PCs and smartphones to simultaneously block distracting elements on both devices, illustrates an early attempt to implement intervention across multiple devices. However, it was an early study that lacked context-aware functionality and required users to manually register distracting elements, limiting it to a relatively rudimentary level. Future research should therefore advance toward system designs that holistically monitor and control the entire cross-device ecosystem surrounding the user, moving beyond control at the level of individual devices.

The structural limitations of single-device interventions are equally confirmed in the approach to the “target behavior” setting. Among all studies, 81% (26/32) target the “total usage time of digital devices” without distinguishing specific activities. Although some studies evaluated interventions targeting specific activities, for instance, Hack Myself (Facebook) [47], the study by Brockmeier et al (Instagram) [21], and SwitchTube (YouTube) [50], such targeted approaches remain a minority (n=6). Moreover, although psychological mechanisms driving overdependence differ fundamentally across activity types, such as the competition or reward cycle in gaming and social comparison and fear of missing out in social networking, studies that design differentiated intervention strategies tailored to the characteristics of various activity types and compare their effects across activity types are lacking.

Within the limited set of activity-specific interventions, video-based services received little attention; only 1 study specifically targeted video-streaming use on YouTube, and none focused on short-form video platforms or algorithmically curated short-video feeds, such as TikTok and YouTube Shorts, despite their rapid growth in recent years. Short-form video possesses uniquely powerful overdependence-inducing design affordances, including algorithm-based personalized feeds and infinite scroll combined with variable-ratio reinforcement. Therefore, existing macro-level control strategies that uniformly block an entire device or app are clearly limited. Future research should actively introduce “feature-level” precision interventions that selectively control only the specific features, such as short-form viewing or recommendation feeds, that induce overdependence, rather than restricting access to the entire app. The absence of empirical studies targeting these powerfully addictive new media forms represents arguably the most urgent research gap in the current DO-DBCI field.

### RQ2: Diversity of Intervention Strategies and Technological Advances

The predominance of self-monitoring indicates that DO-DBCIs have primarily sought to support behavior change by making users’ digital-use patterns visible. The high frequency of nudge and friction- or restriction-based interventions is attributed to the ease of their technical implementation and clarity of theoretical grounding. These intervention strategies can be implemented relatively easily through simple interface modifications or basic system tools (eg, shortcut automation) without technologically complex AI or inference algorithms. Furthermore, as examined in the theoretical background in RQ3: Theoretical Foundations section, these strategies may provide a middle ground, capable of effectively interrupting the “unconscious habitual usage” that constitutes the core mechanism of overuse, while mitigating the extreme resistance provoked by coercive forms of complete blocking.

Many intervention systems analyzed in this review exhibit a clear tendency to rely on uniform, one-size-fits-all static rules, such as “limit to one hour per day” or “unconditional blocking upon specific app launch.” However, behavioral science research indicates that human behavior and overdependence mechanisms vary widely according to individual psychological characteristics such as motivation level and impulsivity [22,59]. Even the same user’s receptivity to an intervention varies greatly depending on context, time, place, emotional state, and the importance of the task at hand. For example, mechanically blocking, even briefly, restorative smartphone use carries a high risk of provoking psychological reactance in users, leading them to abandon the system [67].

Therefore, future research could explore JITAI-based approaches that dynamically adjust intervention timing and intensity according to the user’s context to support sustained behavior change beyond mere usage suppression [16]. JITAI aims to provide “the right type of support, at the right moment, only when needed,” and the most critical technical means for successfully implementing this is context-awareness technology [68].

However, 2 major technical barriers have historically prevented such intelligent interventions from being actively pursued in the literature analyzed. The first is the complexity of contextual data processing. Designing algorithms that connect fragmented data, location, environmental sensors, and device usage patterns to real-time intervention logic is inherently challenging. The second is the limitation of obtaining sufficient training data. Building personalized predictive models requires long-term observation of user behavior patterns, but a substantial proportion of the analyzed studies (approximately 60%) were limited to short-term experiments of 4 weeks or less, making it difficult to obtain the time series data necessary for model refinement.

Overcoming these barriers will require advancement of the technical framework alongside a fundamental change in research design. From an algorithmic perspective, there is a need to introduce a contextual multiarmed bandit framework based on RL to systematize the optimal decision-making structure according to user context [69]. Furthermore, to address the data scarcity problem, a strong case can be made for standardization of longitudinal research designs that extend beyond the current short-term observation-centered approach to include a minimum of 4 weeks intervention and 1 month or more postintervention follow-up [66].

Despite these constraints and challenges, several pioneering studies have recently demonstrated the potential of JITAI through integrated advanced technology stacks. Time2Stop [36] embodied JITAI by learning individual contexts and predicting optimal timing through ML-based adaptive user modeling, and coupled this with a human-in-the-loop (HITL) structure that incorporates user feedback into the AI decisions, attempting to supplement the technical limitations of automated systems with users’ subjective intentions and to enhance acceptability [70]. Jang et al [37] also demonstrated technological progress by computing dynamic personalized incentives according to individual response probabilities through RL.

As intelligent systems grow more sophisticated, XAI becomes a core element, even though the sole pioneering case in the reviewed literature that introduced XAI [36] found that comprehensive Shapley additive explanations (SHAP)–based explanations sometimes caused confusion. XAI that transparently explains why an intervention occurred not only enhances user trust in the system [71] but can also strengthen intrinsic motivation by enabling users to understand their own behavioral patterns. In sum, the technical combination of context-awareness supported by ML, HITL, XAI, and related technologies will be the essential solution for DO-DBCI to evolve from a simple regulatory tool into an intelligent personalized system that deeply understands users’ daily lives and helps them self-regulate.

### RQ3: Current Status of Theoretical Foundations and Systematic Mapping to Intervention Design (Theory-Intervention Mapping)

DO-DBCI draws on more than 20 diverse theories and models, including dual process theory, nudge theory, self-regulation theory, and CBT. This illustrates that digital overuse is a multifaceted phenomenon in which cognitive, motivational, social, and behavioral dimensions are complexly intertwined. However, for these multidisciplinary theories to move beyond declaratory use and drive genuine behavior change, a “theory–intervention mapping” process is essential. This would allow the core constructs of theories to be rigorously translated into specific UI or user experience (UX) elements of the system. Only when theory and system design are clearly connected is it possible to explain through which psychological mechanism a specific technical feature induced behavior change, and to attribute and verify the causes of intervention success or failure precisely [72].

Table 3 presents representative theory-to-design mappings for which the reported design rationale allowed a named theoretical foundation or framework, a core construct or mechanism, and an operationalized UI or UX feature to be traced. These mappings illustrate how theoretical ideas were translated into intervention features as reported by the original authors; they do not indicate intervention effectiveness or methodological quality. For example, TypeOut [24] operationalized dual process theory through a typing-based unlock process intended to interrupt automatic app entry and create a pause for deliberation. Its personalized value-affirmation and action statements represented a separate operationalization of self-affirmation theory. GoldenTime [32] linked self-regulatory and behavioral-economic mechanisms to system-defined hourly timeboxes and loss-framed credit deductions when usage-restriction goals were not met. Together with the other mappings in Table 3, these examples demonstrate the range of ways in which theoretical constructs were translated into specific UI or UX features.

**Table 3:** Representative mappings of theoretical foundations to operationalized UI and user experience features.

Theory or model	Core construct or mechanism	Operationalized design feature	Exemplar study
Behavioral economics	Loss aversion and endowment effect	Pre-endowed gold in the loss condition, with 500 gold deducted after each failed hourly timebox	Park et al [32] (GoldenTime)
Commitment device	Voluntarily binding future behavior to overcome conflict between long-run goals and short-run immediate gratification	Self-defined daily smartphone-use limit with system-enforced weak or strong lockout after goal violation	Kim et al [17] (GoalKeeper)
Dual process theory	Shift from impulsive system 1 toward deliberative system 2 control	Typing-based unlock creates a micro-boundary before target-app access, prompting self-reflection or judgment	Xu et al [24] (TypeOut)
ERG^a^ theory	Relatedness and growth needs	Understanding or comforting for relatedness; evoking or scaffolding habits for growth	Wu et al [55] (MindShift)
Expectancy-value theory	Time or effort cost lowers the net value of a behavioral choice and supports cost-benefit evaluation	Mandatory number-entry lockout with varying workloads before target-app access	Kim et al [23] (LocknType)
Fogg behavior model	Motivation-ability-prompt determinants of habitual use	Disable notifications, reduce phone accessibility, and use greyscale to reverse these determinants	Olson et al [52]
Goal-setting theory	Specific and challenging goals; frequent feedback	Hourly <10-min smartphone-use micromissions with real-time progress, incentive feedback, and threshold notifications	Jang et al [37] (WellbeingWallet)
Health action process approach	Self-efficacy and planning to translate behavioral goals into action	Self-efficacy BCTs^b^ plus action or coping planning exercises specifying when, where, and how to use the smartphone	Keller et al [33] (Not Less But Better)
Mindfulness	Present-moment awareness of context, intentions, and purpose	Unlock-triggered reflection on the purpose of current smartphone use or the intended real-world activity after use	Terzimehić et al [54] (MindPhone)
Nudge theory	Behavior steering while preserving the user’s freedom of final choice	Subtle repeating vibration after the target-app usage limit is exceeded, without blocking app access	Okeke et al [20]
Persuasive systems design model	Self-monitoring; customization	Usage-monitoring feedback and user-configurable wallpaper elements	Nwagu and Orji [19] (Chai Wallpaper)
Self-affirmation theory	Protection of self-integrity through reflection on personally important values	Personalized value-affirmation and action sentences typed before entering the target app	Xu et al [24] (TypeOut)
Self-regulation theory	Self-observation and self-judgment against a desired usage standard	System-driven hourly timeboxes with continuous usage tracking, success or failure evaluation, real-time feedback, and usage dashboard	Park et al [32] (GoldenTime)
Social cognitive theory	Social learning and self-judgment through observation of normative behavior	Shared limiting statistics and within- or between-group rankings supporting comparison and competition	Ko et al [29] (NUGU)

^a^ERG: existence, relatedness, and growth.

^b^BCT: behavior change theory.

Across the full corpus, however, the presence and reporting of such theory-to-design links varied. Of the 32 studies, 12 (38%) did not report a named theoretical foundation or framework as an explicit rationale for intervention design [18,21,25-28,30,34,48,49,51,58]. Among studies that did report a theoretical basis, the complete pathway from theory to construct to operationalized feature was not always fully articulated. In some reports, both a theoretical foundation and relevant intervention components were identifiable, but the hypothesized mechanism connecting them remained implicit or was only briefly described. For example, in the Chai Wallpaper study [19], self-regulation theory was explicitly cited as supporting the choice of a persistent visual-feedback design, but the specific self-regulatory construct linking the theory to the dynamic wallpaper features was not clearly specified. Viewed through the lens of theory-intervention mapping, this incomplete reporting constitutes a theory-to-design translation gap. Importantly, this finding concerns the transparency of theoretical reporting rather than the quality or effectiveness of the interventions. More explicit specification of these links could clarify hypothesized mechanisms, facilitate comparison and replication of design strategies, and support more precise interpretation of how intervention components may contribute to observed outcomes [73]. This structural disconnect between theory and design makes it impossible to objectively verify why an intervention was effective or why it failed. For DO-DBCI research to achieve a qualitative leap, future research should move beyond merely listing theories and explicitly articulate how theoretical constructs inform intervention design. The academic community has long pursued attempts to map “capability-opportunity-motivation” and intervention techniques through the behavior change wheel (BCW) framework proposed by Michie et al [74,75]. However, as the BCW was designed for general health behavior intervention, it has limitations in adequately reflecting the rapidly changing digital context and technical complexity of DO-DBCI [76].

Accordingly, there is an urgent need to introduce a sophisticated design framework that reflects the specificities of DO-DBCI. Such a next-generation framework should include, as a core element, the “design rationale” that transparently demonstrates, beyond simply which theory was borrowed, how the core constructs of the selected theory have been transformed (translated) into specific technical mechanisms of the system. For example, it should explicitly report how the psychological stimuli proposed by a given theory are reflected in the decision-making logic of adaptive algorithms [16], or how they are communicated to users through the XAI interface to stimulate intrinsic motivation [71]. In sum, when a robust causal chain—“mechanism underlying digital overuse→theoretical foundation→core construct→intervention strategy→operationalized design feature”—is established, DO-DBCI will be able to evolve into an intelligent personalized system that simultaneously possesses academic rigor and empirical effectiveness.

### RQ4: Bias in Target Populations and the Need for Expansion

DO-DBCI research is structurally concentrated on university students and young adults, reflecting a methodological tendency arising from differences in research accessibility and recruitment infrastructure. The fact that 80% of the included studies used on-campus communities as recruitment channels directly supports this interpretation. Dependence on convenience samples and overrepresentation of specific age groups are structural problems that have been repeatedly recognized across psychology and behavioral science [77], and the same pattern is reproduced in the DO-DBCI field.

The limited evidence base for adolescent populations is interpreted as a particularly important gap. Adolescence is a period of developing self-regulation capacity and socioemotional development, in which digital use is closely connected to academic performance, sleep, peer relationships, and family interactions [78-80]. Furthermore, because digital use by adolescents is formed within an ecological context different from that of adults, including school norms, parental mediation and supervision, and educational environments, intervention mechanisms validated for adult populations may not have the same ecological appropriateness and effectiveness in adolescents.

However, expanding adolescent research is not simply about increasing the sample size. The limited accumulation of adolescent research may have been influenced by the structural constraints of recruitment and consent procedures. Whereas adult university students have a comparatively systematized research participation infrastructure, such as on-campus communities based on adult autonomy, research targeting minors is often required to undergo guardian permission and adolescent assent procedures. In school-based research in particular, active parental consent approaches have been reported to be associated with lower participation rates and sample bias [81]. Adolescents may also perceive the parental consent process itself as a barrier to research participation [82], and the factors and processes of dropout from screening through enrollment in digital or remote research contexts have been systematically analyzed [83]. As DO-DBCI often involves the collection of highly privacy-sensitive data such as app usage logs, issues of nonresponse and sample representativeness may be further compounded [84]. The tension between parental monitoring demands and adolescent autonomy may create additional attrition conditions at the stages of participation continuation and actual log provision [85,86].

Future DO-DBCI research should therefore include adolescents as a priority population and adopt the following methodological design shifts. First, a phased consent procedure that simultaneously reflects parental consent and adolescent autonomy is necessary. Second, privacy-preserving data collection designs, collecting device-level summary statistics or categorized usage indicators rather than raw logs, should be considered. In particular, differential privacy-based approaches that reduce individual-level information exposure while enabling group-level statistical estimation in local or distributed environments represent a technical option worth exploring in digital behavior research involving adolescents [87].

The low proportion of general user populations also warrants separate consideration. General user samples can potentially contain high within-group heterogeneity in terms of age, occupation, digital literacy, usage purpose, and risk level; therefore, treating them as a single homogeneous group may obscure differential intervention responses behind average effects.

Future research should therefore focus on designing sample frames through previous stratification rather than simply increasing the number of general user samples. For example, setting minimum common stratification variables based on usage motivation (work, academic, or leisure), problem level (general use vs high-risk use), device ecology (smartphone-centric vs multidevice), and digital literacy level can simultaneously enhance comparability across subsequent studies and practical applicability.

Although the pooled gender distribution appeared approximately balanced among studies reporting gender, incomplete reporting and substantial study-level imbalances limited the systematic assessment of gender representativeness across the DO-DBCI evidence base. This issue may be particularly relevant for activity-specific interventions, as previous research suggests that gender-related patterns of problematic digital use can vary across activities. For example, internet gaming disorder has been reported more frequently among males, whereas social media addiction has been reported more frequently among females [88]. Therefore, when an intervention is evaluated using a sample substantially dominated by one gender, caution is warranted when generalizing the findings to users of the underrepresented gender.

The relatively small sample sizes of many DO-DBCI studies further constrain the examination of gender-related heterogeneity in intervention effects. Given the median overall sample size of approximately 46 participants, dividing participants into gender subgroups would often result in small and potentially unequal subgroup sizes, which may provide limited statistical power for reliably detecting gender-related differences in intervention effects. This limitation may become more pronounced when additional demographic characteristics, such as age, are considered simultaneously. Future DO-DBCI studies should therefore ensure consistent demographic reporting and, where gender-specific effects are theoretically relevant, incorporate appropriate recruitment strategies and sample-size planning to support prespecified subgroup analyses.

### RQ5: Research Design, Measurement, and Long-Term Effects

The analysis confirmed that most DO-DBCI studies adopt short-term designs of 4 weeks or less. Although short-term interventions offer practical advantages of high feasibility and low operational burden in real environments, they are accompanied by structural limitations in the ability to distinguish whether changes are maintained after the intervention ends, or whether effects diminish over time and relapse occurs. Since digital overuse is not simply a matter of behavioral frequency but rather the result of complex intertwining of habitual routines, reward structures, and emotional factors, a reduction in usage within the intervention period cannot effectively translate into a restructuring of long-term behavioral patterns.

Furthermore, digital usage problems vary in terms of individual triggers and contexts (time of day, location, task burden, and emotional state), and compliance with or resistance to interventions also varies greatly. Therefore, heuristic-based interventions that apply the same rules uniformly struggle to reflect individual differences precisely. Addressing digital overuse in a sustainable manner, given its high within-person and between-person heterogeneity, requires AI- or ML-based personalized approaches that can adaptively adjust intervention timing, intensity, and form in response to user context and response data. However, since such approaches require data for personalized learning, a time-axis design that ensures sufficient training data in the baseline and intervention stages should be concurrently supported.

Although many studies set a baseline before the intervention, measuring it simply as a fixed period can create representativeness problems. Personal digital usage is sensitive to external factors such as day-of-week patterns, academic and work deadlines, and vacations. Short baselines may therefore fail to adequately represent the typical patterns of an individual, which can affect the validity of subsequent change estimates. In particular, when AI- or ML-based interventions use baseline data as an initial learning period for personalized predictive models or policies, limited observations may prevent adequate capture of within-person variability and contextual diversity, constraining the stability of personalization performance. Therefore, future research should not only extend the baseline period itself but also strengthen the reliability of the reference point through designs such as (1) reporting baseline variability and whether it has stabilized, and (2) multi–time point measurement.

In the intervention stage as well, the duration should be combined with explicit reporting of the learning approach. If an AI or ML model is applied in a fixed state, having been trained during baseline, a longer intervention period does not automatically improve personalization quality. Conversely, if the intervention includes periodic retraining for model updates, the accumulation of individual response data over longer observation periods increases the likelihood that personalization becomes more precise. Future research should therefore explicitly state whether AI- or ML-based interventions (1) rely primarily on baseline-based pre-training, or (2) perform adaptive learning during the intervention and separately evaluate how the personalization implementation approach affects the effect size and maintenance effects.

Additionally, follow-up designs and verification data for validating the sustainability of intervention effects are insufficient. The fact that only a minority of studies conducted follow-up, and that those who did predominantly reported short-term 1-2 week tracking, suggests that current evidence can capture short-term changes immediately after intervention conclusion, but has insufficiently verified whether changes persist after time passes, or whether usage patterns revert to preintervention levels under conditions where external control is weakened. In particular, as autonomous choices and environmental inducements are again strengthened after the intervention ends, interpreting maintenance effects is difficult in studies without follow-up evaluation.

To compensate for these limitations, future DO-DBCI research needs to structure an evaluation schedule rather than simply extending intervention duration. It is advisable to design studies that include at least 1 delayed postevaluation, with multi–time point tracking where possible, to evaluate short-term change and maintenance effects. Furthermore, to enhance the interpretability of maintenance effects, it is desirable to report immediate effect, maintenance effect, and relapse indicators separately; to combine objective usage logs with self-report measures; and to present participant retention, compliance, dropout rate, and reasons for dropout. When such design refinements are implemented in parallel, DO-DBCI can establish itself as an empirically grounded intervention capable of demonstrating sustainable digital well-being in real environments, going beyond the observation of short-term behavioral suppression effects.

### Integrated Recommendations for Future DO-DBCI Research

Future DO-DBCI research should evolve into an “intelligent behavior change science” in which theory and technology are organically combined, going beyond the mere development of behavioral suppression tools. The primary objective is the establishment of a vertical alignment following the chain: “mechanism underlying digital overuse→theoretical foundation→core construct→intervention strategy→operationalized design feature.” To resolve the fragmented connectedness between theory and design identified in this review, researchers should explicitly report how the active ingredients of the selected theory have been translated into specific functional elements or interfaces of the system. This transparent sharing of “design rationale” will serve as the core criterion for demonstrating the academic validity of a given intervention, regardless of technical complexity.

A clearer theory-to-design pathway can inform not only which intervention components are implemented but also when and under what conditions they should be delivered [89]. Accordingly, future research could explore context-sensitive and adaptive approaches, including the JITAI framework, particularly where users’ needs or receptivity vary over time. What matters here is not just the introduction of complex algorithms, but rather how the system incorporates users’ actual response data and feedback to adjust the timing and intensity of interventions flexibly. To this end, the system should simultaneously secure adaptability and user acceptance by combining a HITL structure that reflects user intentions, while establishing design principles that transparently provide the basis for interventions to users, strengthening self-awareness and intrinsic motivation.

In addition, an ecosystemic expansion of the scope and target of interventions is required. To counter the “balloon effect” arising from single device-centered control, a cross-device integrated architecture that encompasses the user’s entire digital environment should be designed. In particular, empirical studies on media-specific interventions for media types with distinctive behavioral induction mechanisms, such as the rapidly proliferating short-form video, urgently need to follow. From a demographic perspective, research populations should be expanded, particularly to adolescents, while privacy-preserving data collection methodologies that account for the complex ecological context in which developmental characteristics, parental supervision rights, and individual autonomy collide could be established.

Finally, standardization of research design should be achieved to raise the level of empirical evidence in DO-DBCI research. This review recommends a research design that includes a minimum of 4 weeks of intervention along with 1 month or more of follow-up observation, taking into account the initial threshold of habit formation at which new behavioral patterns become internalized [90], and enabling rigorous verification of users’ autonomous self-regulation capacity after external system control is removed [66]. This serves as an indispensable methodological standard for distinguishing between transient changes immediately after intervention and genuine long-term behavior modification.

### Limitations

This scoping review systematically mapped the research landscape of the DO-DBCI field and identified key research gaps. However, the following limitations were noted.

First, there are areas where the scope of literature covered by this review is constrained by the level of accumulated research in the field itself. In particular, problematic use of short-form video platforms, represented by TikTok, is among the most rapidly growing forms of digital overuse. Yet DO-DBCI studies directly targeting this behavior had accumulated only to an extremely limited extent by the time of this search, resulting in inadequate mapping of this area.

Furthermore, although AI- or ML-based adaptive interventions represent the most prominent technical direction within the field, the number of relevant studies remains small, making systematic comparative analysis of this intervention type limited. Similarly, given that approaches using LLMs as intervention tools represent a noteworthy technical direction within the field, the accumulation of relevant empirical studies remains in its early stages, and studies meeting the inclusion criteria of this review were insufficient. De Russis et al [91] exploratorily confirmed that LLM-based chatbots can propose personalized strategies for smartphone overuse personas, but empirical validation of actual intervention effects has not yet been achieved. Systematic examination of this area remains a task for subsequent reviews.

As DO-DBCI is a field that is rapidly evolving alongside AI and mobile sensing technologies, some of these limitations partially reflect that the field itself is still in an early stage. The fact that the final set of included studies was limited to 32 can be understood in this context, and caution is required in generalizing some analytical results. As the field matures and related research accumulates, updated reviews will be necessary.

### Conclusion

This scoping review analyzed 32 DO-DBCI studies across 5 dimensions—target devices and behaviors, intervention strategies, theoretical foundations, target populations, and intervention duration and sustainability of effects.

The analysis confirms that the DO-DBCI field has achieved remarkable technological advances over the past decade. Leading cases, such as TypeOut and GoldenTime, which rigorously translate dual process theory and self-regulation theory into interface design; Time2Stop, which integrated ML-based adaptive modeling with a HITL structure; and PomodoLock, which pioneered multidevice environments, empirically demonstrated the direction in which the field should advance. However, alongside these pioneering cases, structural challenges across the field were also identified. Most studies remained confined to uniform control targeting single devices and total usage time, failing to encompass the multidevice ecosystem and the characteristics of new media types, such as short-form video. Studies designed without theoretical grounding accounted for 38% (12/32), and even studies that cited theory frequently failed to explicitly describe the logical process by which core constructs were connected to specific functions. Additionally, structural limitations existed in the generalizability and sustainability verification of intervention effects due to the dependence on convenience samples centered on university students and young adults, and the concentration of short-term designs of 4 weeks or less.

To overcome these challenges, this review proposes an integrated direction for the qualitative advancement of DO-DBCI research. The most urgent priority is establishing vertical alignment along the structured theory-to-design pathway proposed in this review. The core of this alignment is explicitly articulating the design rationale that shows how the active ingredients of the selected theory have been translated into the specific functions and interfaces of the system. Building on this theoretical foundation, the JITAI paradigm, XAI, and the HITL structure each offer mechanisms for aligning interventions with users’ real‑time contexts, decision transparency, and subjective intentions. These approaches incorporate users’ subjective intentions into automated systems and could be introduced in an integrated manner. Additionally, expansion to a cross-device architecture beyond single-device control, broadening of research populations to include diverse groups, particularly adolescents, and standardization of research designs incorporating a minimum of 4 weeks of intervention and 1 month or more of follow-up observation should be pursued in parallel.

The comprehensive research landscape of the DO-DBCI field and the critical recommendations presented in this review will serve as a useful academic compass for researchers and designers going forward, guiding them in constructing intelligent digital well-being systems in which theory and technology are organically combined, moving beyond fragmented approaches.

## Data Availability

All data generated and analyzed in this scoping review are provided in the manuscript and Multimedia Appendices. No individual-level participant data were collected. Additional review materials are available from the corresponding author upon reasonable request.

## Appendix

Multimedia Appendix 1 PRISMA-ScR Checklist.

Multimedia Appendix 2 Database search details.

Multimedia Appendix 3 Summary of selected studies on digital well-being interventions.

## Abbreviations

ACT: acceptance and commitment therapy
BCW: behavior change wheel
CBT: cognitive behavioral therapy
DBCI: digital behavior change intervention
DO-DBCI: digital overuse-targeted digital behavior change intervention
DSCT: digital self-control tool
ERG: existence, relatedness, and growth
ICD-11: International Classification of Diseases 11th Revision
HCI: human-computer interaction
HITL: human-in-the-loop
JITAI: just-in-time adaptive intervention
ML: machine learning
OS: operating system
PRISMA-ScR: Preferred Reporting Items for Systematic Reviews and Meta-Analyses Extension for Scoping Reviews
PSU: problematic smartphone use
RL: reinforcement learning
RQ: research question
SHAP: Shapley additive explanations
TTM: transtheoretical model
UX: user experience
XAI: explainable AI
