# Supplementary material for: Digital Behavior Change Interventions for Digital Overuse: Scoping Review of Current Approaches and Emerging AI-Enabled Systems
Source: J Med Internet Res. 2026 Sep 23;28:e101150. doi: 10.2196/101150 (PMC13646779; doi:10.2196/101150)
Supplement: Supplementary file 2 [file jmir_v28i1e101150_app2.docx]

**Multimedia Appendix 2**. Database search details

| **DB** | **Search Query** | **Number of Studies** |
| --- | --- | --- |
| **Web of Science** | TS=("computer usage" OR "digital wellbeing" OR "digital overuse" OR "digital distraction" OR "digital dependence" OR "gaming disorder" OR "internet overuse" OR "mobile phone overuse" OR "mobile device overuse" OR "mobile distraction" OR "off-task usage" OR "smartphone overuse" OR "smartphone usage" OR "smartphone use" OR "smartphone non-use" OR "smartphone app use" OR "smartphone addiction" OR "smartphone distraction" OR "social media usage" OR "time-wasting websites" OR "time online" ) AND TS=("behavior change" OR "behavior restriction" OR "intervention" OR "limiting behavior" OR "persuasive technology" OR "self-control" OR "self-discipline" OR "self-monitoring" OR "self-regulation" OR "time application") * Timespan: 2008-01-01 to 2025-12-31 | 2029 |
| **ACM Digital Library** | [[Abstract: "behavior change"] OR [Abstract: "behavior restriction"] OR [Abstract: "intervention"] OR [Abstract: "limiting behavior"] OR [Abstract: "persuasive technology"] OR [Abstract: "self-control"] OR [Abstract: "self-discipline"] OR [Abstract: "self-monitoring"] OR [Abstract: "self-regulation"] OR [Abstract: "time application"]] AND [[Abstract: "computer usage"] OR [Abstract: "digital wellbeing"] OR [Abstract: "digital overuse"] OR [Abstract: "digital distraction"] OR [Abstract: "digital dependence"] OR [Abstract: "gaming disorder"] OR [Abstract: "internet overuse"] OR [Abstract: "mobile phone overuse"] OR [Abstract: "mobile device overuse"] OR [Abstract: "mobile distraction"] OR [Abstract: "off-task usage"] OR [Abstract: "smartphone overuse"] OR [Abstract: "smartphone usage"] OR [Abstract: "smartphone use"] OR [Abstract: "smartphone non-use"] OR [Abstract: "smartphone app use"] OR [Abstract: "smartphone addiction"] OR [Abstract: "smartphone distraction"] OR [Abstract: "social media usage"] OR [Abstract: "time-wasting websites"] OR [Abstract: "time online"]] AND [E-Publication Date: (01/01/2008 TO 12/31/2025)] | 125 |
| **IEEE Xplore** | (("Abstract":"behavior change" OR "Abstract":"behavior restriction" OR "Abstract":"intervention" OR "Abstract":"limiting behavior" OR "Abstract":"persuasive technology" OR "Abstract":"self-control" OR "Abstract":"self-discipline" OR "Abstract":"self-monitoring" OR "Abstract":"self-regulation" OR "Abstract":"time application" OR "Document Title":"behavior change" OR "Document Title":"behavior restriction" OR "Document Title":"intervention" OR "Document Title":"limiting behavior" OR "Document Title":"persuasive technology" OR "Document Title":"self-control" OR "Document Title":"self-discipline" OR "Document Title":"self-monitoring" OR "Document Title":"self-regulation" OR "Document Title":"time application") AND ("Abstract":"computer usage" OR "Abstract":"digital wellbeing" OR "Abstract":"digital overuse" OR "Abstract":"digital distraction" OR "Abstract":"digital dependence" OR "Abstract":"gaming disorder" OR "Abstract":"internet overuse" OR "Abstract":"mobile phone overuse" OR "Abstract":"mobile device overuse" OR "Abstract":"mobile distraction" OR "Abstract":"off-task usage" OR "Abstract":"smartphone overuse" OR "Abstract":"smartphone usage" OR "Abstract":"smartphone use" OR "Abstract":"smartphone non-use" OR "Abstract":"smartphone app use" OR "Abstract":"smartphone addiction" OR "Abstract":"smartphone distraction" OR "Abstract":"social media usage" OR "Abstract":"time-wasting websites" OR "Abstract":"time online" OR "Document Title":"computer usage" OR "Document Title":"digital wellbeing" OR "Document Title":"digital overuse" OR "Document Title":"digital distraction" OR "Document Title":"digital dependence" OR "Document Title":"gaming disorder" OR "Document Title":"internet overuse" OR "Document Title":"mobile phone overuse" OR "Document Title":"mobile device overuse" OR "Document Title":"mobile distraction" OR "Document Title":"off-task usage" OR "Document Title":"smartphone overuse" OR "Document Title":"smartphone usage" OR "Document Title":"smartphone use" OR "Document Title":"smartphone non-use" OR "Document Title":"smartphone app use" OR "Document Title":"smartphone addiction" OR "Document Title":"smartphone distraction" OR "Document Title":"social media" OR "Document Title":"time-wasting websites" OR "Document Title":"time online")) * Filters Applied: 2008 - 2025 | 166 |
| **Pubmed** | ("behavior change"[Title/Abstract] OR "behavior restriction"[Title/Abstract] OR "intervention"[Title/Abstract] OR "limiting behavior"[Title/Abstract] OR "persuasive technology"[Title/Abstract] OR "self-control"[Title/Abstract] OR "self-discipline"[Title/Abstract] OR "self-monitoring"[Title/Abstract] OR "self-regulation"[Title/Abstract] OR "time application"[Title/Abstract]) AND ("computer usage"[Title/Abstract] OR "digital wellbeing"[Title/Abstract] OR "digital overuse"[Title/Abstract] OR "digital distraction"[Title/Abstract] OR "digital dependence"[Title/Abstract] OR "gaming disorder"[Title/Abstract] OR "internet overuse"[Title/Abstract] OR "mobile phone overuse"[Title/Abstract] OR "mobile device overuse"[Title/Abstract] OR "mobile distraction"[Title/Abstract] OR "off-task usage"[Title/Abstract] OR "smartphone overuse"[Title/Abstract] OR "smartphone usage"[Title/Abstract] OR "smartphone use"[Title/Abstract] OR "smartphone non-use"[Title/Abstract] OR "smartphone app use"[Title/Abstract] OR "smartphone addiction"[Title/Abstract] OR "smartphone distraction"[Title/Abstract] OR "social media usage"[Title/Abstract] OR "time-wasting websites"[Title/Abstract] OR "time online"[Title/Abstract])  * Filters: from 2002 - 2025 | 940 |
